# Insomnia Symptoms in a Swiss Population‐Based Cohort: Prevalence, Clinical Correlates and Polysomnographic Alterations

**DOI:** 10.1111/jsr.70376

**Published:** 2026-06-04

**Authors:** Isabel Ericson, Geoffroy Solelhac, Théo Imler, Kari Lambing, Magdy Younes, Pedro Marques‐Vidal, Peter Vollenweider, Marie‐Pierre Françoise Strippoli, Martin Preisig, Raphaël Heinzer, Nicola A. Marchi

**Affiliations:** ^1^ Center for Investigation and Research on Sleep, Department of Medicine Lausanne University Hospital and University of Lausanne Lausanne Switzerland; ^2^ YRT Limited Winnipeg Manitoba Canada; ^3^ Department of Psychology Brock University St Catharines Ontario Canada; ^4^ Department of Medicine University of Manitoba Winnipeg Manitoba Canada; ^5^ Department of Medicine, Internal Medicine Lausanne University Hospital and University of Lausanne Lausanne Switzerland; ^6^ Psychiatric Epidemiology and Psychopathology Research Center, Department of Psychiatry Lausanne University Hospital and University of Lausanne Prilly Switzerland

**Keywords:** EEG spectral analysis, epidemiology, hypnotics, odds ratio product, polysomnography, sleep microstructure

## Abstract

This study assessed the prevalence, clinical correlates and polysomnographic features of insomnia symptoms in a population‐based cohort (HypnoLaus, Lausanne, Switzerland). Data were obtained from 3947 participants (mean age 57.5 ± 10.5 years; range 40–82; 52.0% female). Insomnia symptoms were defined as difficulty initiating or maintaining sleep ≥ 3 nights/week during the past month or regular hypnotic use. Independent correlates of insomnia symptoms were examined using logistic regression. In the subset of 1718 participants who underwent polysomnography (performed 1.1 ± 0.9 years after insomnia symptom assessment), associations between insomnia symptoms and sleep variables were examined using linear regression models adjusted for age, sex, depression, anxiety and psychotropic use. Insomnia symptoms affected 32.0% of participants, and 4.2% of the total sample reported regular hypnotic use. Independent correlates included older age, female sex, lower education, dyslipidaemia, lower likelihood of current smoking, excessive alcohol consumption, depression, anxiety, psychotropic use and daytime sleepiness. Compared with those without symptoms, participants with insomnia showed shorter total sleep time, longer sleep onset latency, longer wake after sleep onset and lower sleep efficiency (all small effect sizes). Sleep microstructure analysis showed higher odds ratio product (ORP) values (in wake, NREM, and N2, and ORP‐9), higher normalised EEG power, higher beta power and higher gamma power (all small effect sizes). Insomnia symptoms are highly prevalent in this population‐based cohort and are linked to multiple sociodemographic and clinical factors. Polysomnographic findings indicate modest sleep macrostructural differences, whereas microstructural alterations, although subtle in magnitude, appeared more consistently associated with insomnia symptoms.

## 1 Introduction

Insomnia is among the most prevalent sleep disorders, with population‐based studies reporting symptoms in 20%–30% of adults and chronic disorder in about 10% (Hafner et al. 2023; Bjorvatn et al. 2023). In addition, the prevalence of insomnia is rising (Pallesen et al. 2014). The mechanisms underlying insomnia, particularly those involving objective sleep physiology, remain poorly understood. Unlike other sleep disorders with defined polysomnographic (PSG) signatures, insomnia is diagnosed by self‐report. Nevertheless, objective measures may provide insight.

Most PSG studies have focused on clinical populations or specific subtypes, such as primary insomnia (Baglioni et al. 2014). While informative, they may not reflect the full spectrum of symptoms in the general population. The limited number of population‐based investigations has yielded inconsistent findings: some report shorter total sleep duration and reduced sleep efficiency in individuals with insomnia compared with those without, while other studies report minimal or no differences (Ma et al. 2021; Kang et al. 2022; Lyu et al. 2020; Lucena et al. 2020). These mixed results support the view that conventional PSG macrostructure metrics, such as total sleep time, sleep efficiency and sleep‐stage proportions, may not fully capture the physiological alterations associated with insomnia.

One proposed mechanism underlying insomnia is cortical hyperarousal, reflected in microstructural EEG alterations that may not be captured by conventional PSG macrostructure. Sleep microstructure studies have frequently reported a shift toward faster, wake‐like activity, including elevated beta and gamma power, or reduced slow activity during NREM sleep, although findings vary by sleep stage, sex and analytic approach (Perlis, Kehr, et al. 2001; Perlis, Smith, et al. 2001; Buysse et al. 2008; Perrier et al. 2015). Beyond spectral changes, insomnia has also been associated with sleep instability, including increased cyclic alternating pattern rate (Parrino et al. 2012) and altered indices of sleep depth and arousability, including higher odds ratio product (ORP) and slower post‐arousal recovery dynamics (ORP‐9) (Younes et al. 2021; Lambing et al. 2025). However, much of the available evidence derives from clinical samples with modest sizes and heterogeneous EEG methodologies, limiting generalisability and potentially contributing to inconsistent findings. This highlights the need for large population‐based investigations examining EEG‐derived microstructural markers of arousal, sleep instability and sleep depth.

This study analysed data from a population‐based sample of adults in Lausanne, Switzerland, who completed the Pittsburgh Sleep Quality Index (PSQI) (Buysse et al. 1989) and underwent at‐home PSG, allowing assessment of both subjective insomnia symptoms and objective sleep metrics. Our objectives were: (1) estimate the prevalence of insomnia symptoms; (2) identify the sociodemographic and clinical correlates; and (3) examine associations between insomnia symptoms and measures of sleep macrostructure and microstructure.

## 2 Methods

### 2.1 Sample

Data came from the CoLaus|PsyCoLaus study, a cohort of community‐dwelling adults in Lausanne, Switzerland, including detailed physical and psychiatric assessments at baseline and three completed follow‐ups (Firmann et al. 2008; Preisig et al. 2009). At each follow‐up, sociodemographic and clinical information were recorded, and participants completed sleep questionnaires including the PSQI. The present analysis focuses on the first follow‐up (2009–2013), yielding 3947 participants with PSQI data, of whom 1718 also had participated in the HypnoLaus sub‐study and underwent one night of at‐home PSG (Figure S1). Exclusion criteria were (1) extreme chronotype (Morningness–Eveningness Questionnaire [MEQ] ≤ 30 or ≥ 70; *n* = 1) (Shahid et al. 2011), and (2) restless legs syndrome (based on the four essential diagnostic criteria; *n* = 637) (Allen et al. 2003). These conditions can produce sleep complaints and nocturnal symptoms that may be misattributed to insomnia, increasing the risk of misclassification. Ethics approval and written consent were obtained. Additional ethical information is available in the Supporting Information.

### 2.2 Insomnia Symptoms

Insomnia symptoms were determined based on data recorded during the first follow‐up of the CoLaus|PsyCoLaus study and were defined using items extracted from the PSQI and the use of hypnotics. Participants were classified as having insomnia symptoms if they reported difficulty initiating sleep (sleep onset latency > 30 min) or nocturnal/early‐morning awakenings occurring ≥ 3 nights/week during the past month. To avoid misclassifying individuals whose symptoms may be pharmacologically controlled at the time of assessment, regular use of hypnotics was retained as an indicator of insomnia symptoms. Regular medication use was assessed by asking participants: “During the past 6 months, have you regularly taken any medications, either with or without a prescription?” Hypnotics were defined as medications coded N05C (“hypnotics and sedatives”) according to the World Health Organization Anatomical Therapeutic Chemical (WHO/ATC) classification (www.whocc.no/atc_ddd_index). Participants who answered affirmatively to medication use and reported N05C medications were classified as regular hypnotic users and categorised as having insomnia symptoms.

### 2.3 Sociodemographic and Clinical Variables

Sociodemographic and clinical variables were recorded at the first follow‐up of the CoLaus|PsyCoLaus study and selected based on their clinical plausibility as being associated with insomnia symptoms. Age, sex and education level (number of years of formal schooling) were recorded. Body mass index (BMI) was calculated as weight divided by height squared (kg/m^2^). Hypertension was defined as blood pressure ≥ 140/90 mmHg and/or use of antihypertensive medication. Diabetes was defined as fasting blood glucose ≥ 7.0 mmol/L and/or use of antidiabetic drugs. Dyslipidaemia was defined as the presence of low high‐density‐lipoprotein cholesterol (< 1.00 mmol/L in males, < 1.29 mmol/L in females), high triglycerides (≥ 2.2 mmol/L) and/or elevated low‐density lipoprotein cholesterol (≥ 4.1 mmol/L, or ≥ 2.6 mmol/L in participants with a history of myocardial infarction, stroke, coronary artery disease, or diabetes). Smoking status was categorised as current smoker vs. never or former smoker. Excessive alcohol consumption was defined as ≥ 14 units per week. Psychiatric disorders, including major depressive disorder and anxiety disorders, were assessed using the French version of Diagnostic Interview for Genetic Studies (Leboyer et al. 1995; Nurnberger et al. 1994), complemented by the anxiety disorder modules from the French version of the Schedule for Affective Disorders and Schizophrenia–Lifetime and Anxiety Disorder version (Leboyer et al. 1991; Endicott and Spitzer 1978). Diagnoses were assigned according to the Diagnostic and Statistical Manual of Mental Disorders‐IV criteria (DSM‐IV‐TR 2000). Depression was defined as the presence of a current episode of major depressive disorder, and anxiety as a current generalised anxiety disorder, agoraphobia, social phobia, or panic disorder. Psychotropic medication use was defined as the regular use of any of the following drug classes according to the WHO/ATC classification: antidepressants (N06A), anxiolytics (N05B), or antipsychotics (N05A). Sleep‐related questionnaires included the PSQI, the Epworth Sleepiness Scale (ESS; subjective daytime sleepiness) and the MEQ (chronotype).

### 2.4 Polysomnography

PSG variables were recorded during the HypnoLaus sub‐study, a mean of 1.1 ± 0.9 years after the assessment of insomnia symptoms and other sociodemographic and clinical variables. Participants underwent one night of home‐based PSG, as previously described (Heinzer et al. 2015; Marchi et al. 2022, 2024). Scoring was performed blinded to insomnia status. Sleep stages and arousals were scored using the American Academy of Sleep Medicine (AASM) 2007 criteria (Iber et al. 2007), while respiratory events were scored following the AASM 2012 guidelines (Berry et al. 2012). Periodic limb movements during sleep (PLMS) were scored according to World Association of Sleep Medicine 2006 recommendations (Zucconi et al. 2006), as performed in previous studies by our group (Haba‐Rubio et al. 2016; Marchi et al. 2023, 2026). Full details on PSG setup, acquisition, scoring and quality control are available in the Supporting Information.

### 2.5 Sleep Macrostructural Metrics

The following sleep macrostructure variables were selected and analysed, as they represent conventional PSG parameters commonly used in both research and clinical practice: total sleep time (TST), sleep onset latency, REM latency, sleep stage proportions, sleep efficiency, wake after sleep onset (WASO), apnea–hypopneaindex (AHI), PLMS index, awakening index (sleep‐to‐wake transitions per hour) and arousal index (arousals per hour). Arousal subtypes included spontaneous, respiratory‐related, respiratory effort‐related (RERAs) and PLMS‐related arousals.

### 2.6 Sleep Microstructural Metrics

Sleep microstructure analysis was performed using EEG signals from the central derivations and included the ORP, as previously described (Younes et al. 2015). ORP is a continuous measure of sleep depth derived from the interaction of powers across multiple frequency bands using Fast Fourier Transform (FFT) analysis. It is expressed on a unitless scale from 0 (very deep sleep) to 2.5 (full wakefulness) and calculated in 3‐s epochs throughout the recording. ORP was computed for wake, each sleep stage and the 9 s following an arousal (ORP‐9) (Younes and Hanly 2016). ORP‐9 reflects the rate at which sleep returns to deeper levels following an arousal (higher values indicate slower progression to deep sleep and greater sleep instability). Additional spectral features from FFT were examined, including interhemispheric coherence of ORP (ORP‐ICC; defined as the intra‐class correlation coefficient quantifying the instantaneous correlation between right and left hemispheric ORP values), normalised EEG power during total recording time (a unitless measure reflecting the overall EEG signal amplitude relative to the cohort average, calculated by dividing individual absolute power in each frequency band by the cohort mean for the band and averaging the ratios when they are approximately uniform across bands) (Younes et al. 2023) and alpha intrusion (defined as the percentage of 3‐s epochs in NREM sleep with alpha power > 30 μV^2^) (Younes et al. 2021). Finally, absolute power (expressed in μV^2^) was calculated for the following bands: delta (0.5–4 Hz), theta (4–8 Hz), alpha (8–12 Hz), sigma (12–14 Hz), beta‐1 (14–20 Hz), beta‐2 (20–35 Hz) and gamma (35–45 Hz).

### 2.7 Statistical Analysis

Descriptive statistics were used to compare participants with and without insomnia symptoms. Independent *t*‐tests were applied for continuous variables, and chi‐squared tests for categorical variables. Multivariable binary logistic regression was performed to examine sociodemographic and clinical characteristics (independent variables) independently associated with insomnia symptoms (dependent variable). All sociodemographic and clinical variables were entered simultaneously into the model based on their clinical plausibility of being associated with insomnia symptoms, regardless of whether they showed significant differences in the univariate analyses. Results are presented as odds ratios (OR) with 95% confidence intervals (CI). Associations between PSG‐derived sleep metrics (dependent variables) and insomnia symptoms (independent variable) were assessed using linear regression models. Models were adjusted for age, sex, current major depressive disorder, current anxiety disorder and psychotropic medication use. Skewed PSG‐derived variables were square‐root or log‐transformed to meet model assumptions, and results were back‐transformed for presentation. Sensitivity analyses were conducted to evaluate potential insomnia × age and insomnia × sex interactions for all PSG variables. Additional sensitivity analyses accounted for differences in study samples, as not all participants underwent PSG, and among those who did, 144 had missing microstructure data. Results are presented as adjusted means with standard errors, as well as unstandardised beta coefficients (B) with 95% CI. Cohen's *d* coefficients were reported to quantify effect size. All tests were two‐sided, and a *p* < 0.05 was considered statistically significant. Analyses were conducted in R (version 4.4.1; R Core Team, 2024). Procedures for handling missing data are described in the Supporting Information.

## 3 Results

### 3.1 Sociodemographic and Clinical Factors

Of 3947 adults (mean age 57.5 ± 10.5 years; range 40–82; 52.0% female), 32.0% had insomnia symptoms. Regular use of hypnotics, which was included in the operational definition of insomnia symptoms, was reported by 4.2% of participants overall and by 12.2% of those with insomnia symptoms.

Compared with participants without insomnia symptoms, those with insomnia symptoms were older, more likely to be female, had fewer years of education, had a higher prevalence of hypertension, were less likely to be current smokers and were more likely to have a current major depressive disorder, current anxiety disorder and to use psychotropic medication (Table 1).

**TABLE 1:** Characteristics of participants with and without insomnia symptoms (*n* = 3947).

	No insomnia symptoms (*n* = 2682; 68.0%)	Insomnia symptoms (*n* = 1265; 32.0%)	Test	*p*
Age, years	56.7 ± 10.4	59.2 ± 10.4	*t* = 7.2	**< 0.001**
Female, *n* (%)	1309 (48.8)	743 (58.7)	*x* ^2^ = 33.5	**< 0.001**
Education, years	13.9 ± 4.2	13.3 ± 3.8	*t* = 4.8	**< 0.001**
BMI, kg/m^2^	26.0 ± 4.4	26.1 ± 4.6	*t* = 0.8	0.427
Hypertension, *n* (%)	1034 (38.6)	565 (44.8)	*x* ^2^ = 13.2	**< 0.001**
Diabetes, *n* (%)	248 (9.3)	126 (10.0)	*x* ^2^ = 0.4	0.527
Dyslipidaemia, *n* (%)	709 (26.4)	368 (29.1)	*x* ^2^ = 2.9	0.081
Current smoker, *n* (%)	657 (21.8)	303 (19.3)	*x* ^2^ = 3.9	**0.048**
Excessive alcohol consumption, *n* (%)	398 (14.8)	211 (16.7)	*x* ^2^ = 2.1	0.148
Current major depressive disorder, *n* (%)	111 (4.9)	94 (8.7)	*x* ^2^ = 18.7	**< 0.001**
Current anxiety disorder, *n* (%)	103 (4.8)	79 (7.9)	*x* ^2^ = 11.2	**< 0.001**
Psychotropic medication, *n* (%)	222 (8.3)	205 (16.2)	*x* ^2^ = 55.2	**< 0.001**
ESS score, points	5.6 ± 3.6	5.7 ± 3.9	*t* = 0.4	0.701
MEQ chronotype			*x* ^2^ = 4.3	0.113
Intermediate, *n* (%)	2151 (89.2)	999 (89.5)		
Evening, *n* (%)	241 (10.0)	100 (9.0)		
Morning, *n* (%)	20 (0.8)	17 (1.5)		

*Note:* Data are presented as mean ± standard deviation or number of participants (%). Data were analysed using independent samples *t*‐tests (*t*) and chi‐squared tests (*x* ^2^). Missing values: BMI (*n* = 36), hypertension (*n* = 8), diabetes (*n* = 13), current smoker (*n* = 6), current major depressive disorder (*n* = 585), current anxiety disorder (*n* = 805), ESS score (*n* = 189), MEQ (*n* = 419). Complete case analysis was performed. Bold text indicates *p* < 0.05.

Abbreviations: BMI, body mass index; ESS, Epworth Sleepiness Scale; MEQ, Morningness–Eveningness Questionnaire.

In the multivariable model, insomnia symptoms were independently associated with older age, female sex, fewer years of education, dyslipidaemia, a lower likelihood of being a current smoker, excessive alcohol consumption, current major depressive disorder, current anxiety disorder, psychotropic medication use and higher ESS scores (Table 2).

**TABLE 2:** Sociodemographic and clinical characteristics associated with insomnia symptoms (*n* = 3947).

	Unadjusted OR (95% CI)	*p*	Adjusted OR (95% CI)	*p*
Age (per 5‐year increase)	1.12 (1.09, 1.16)	**< 0.001**	1.12 (1.08, 1.16)	**< 0.001**
Female sex	1.49 (1.30, 1.71)	**< 0.001**	1.50 (1.29, 1.75)	**< 0.001**
Education (per 1‐year increase)	0.96 (0.94, 0.98)	**< 0.001**	0.97 (0.96, 0.99)	**0.003**
BMI (per 1‐kg/m^2^ increase)	1.01 (0.99, 1.02)	0.418	0.99 (0.98, 1.01)	0.526
Hypertension	1.29 (1.12, 1.47)	**< 0.001**	1.10 (0.93, 1.29)	0.260
Diabetes	1.08 (0.86, 1.35)	0.514	0.90 (0.70, 1.16)	0.422
Dyslipidaemia	1.14 (0.99, 1.33)	0.075	1.22 (1.04, 1.44)	**0.018**
Current smoker	0.85 (0.72, 1.00)	0.053	0.79 (0.66, 0.94)	**0.009**
Excessive alcohol consumption	1.15 (0.96, 1.38)	0.136	1.35 (1.11, 1.64)	**0.003**
Current major depressive disorder	1.90 (1.43, 2.52)	**< 0.001**	1.52 (1.12, 2.08)	**0.008**
Current anxiety disorder	1.71 (1.27, 2.31)	**< 0.001**	1.55 (1.14, 2.10)	**0.006**
Psychotropic medication	2.14 (1.75, 2.63)	**< 0.001**	1.76 (1.42, 2.19)	**< 0.001**
ESS score (per 1‐point increase)	1.00 (0.99, 1.02)	0.687	1.02 (1.00, 1.04)	**0.031**
MEQ chronotype				
Intermediate (ref.)	1.00	—	1.00	—
Evening	1.81 (0.94, 3.46)	0.074	1.97 (1.00, 3.88)	0.051
Morning	0.89 (0.70, 1.14)	0.360	0.85 (0.66, 1.10)	0.211

*Note:* Data were analysed using binomial logistic regression with insomnia symptoms as the dependent variable. Unadjusted OR were calculated for each predictor separately, whereas adjusted OR were derived from a multivariable model including all predictors listed in the table. Missing data were handled using multiple imputation. Results are presented as odds ratios (OR) with 95% confidence intervals (CI). Bold text indicates *p* < 0.05.

Abbreviations: BMI, body mass index; ESS, Epworth Sleepiness Scale; MEQ, Morningness–Eveningness Questionnaire.

### 3.2 Sleep Macrostructure

Compared to participants without insomnia symptoms, those with insomnia symptoms showed shorter TST (−8.8 min), longer sleep onset latency (+1.4 min), longer WASO (+7.0 min) and lower sleep efficiency (−1.6%). All significant differences were small in magnitude (Table 3).

**TABLE 3:** Sleep macrostructure variables associated with insomnia symptoms (*n* = 1718).

	No insomnia symptoms (*n* = 1144; 66.6%)	Insomnia symptoms (*n* = 574; 33.4%)	B (95% CI)	*p*	Cohen's *d*
TST (min)	403.1 ± 2.0	394.4 ± 2.9	−8.8 (−15.8, −1.8)	**0.014**	0.13
Sleep onset latency (min)^b^	9.8 ± 0.3	11.1 ± 0.5	1.4 (0.2, 2.2)	**0.022**	0.12
REM latency (min)^a^	87.1 ± 1.4	88.8 ± 2.1	1.7 (−3.3, 6.7)	0.493	0.03
Sleep efficiency (%)	85.3 ± 0.3	83.7 ± 0.4	−1.6 (−2.6, −0.6)	**0.002**	0.16
WASO (min)	71.5 ± 1.5	78.5 ± 2.1	7.0 (1.9, 12.1)	**0.007**	0.14
Stage N1 (%)	12.0 ± 0.2	11.9 ± 0.3	−0.1 (−0.7, 0.6)	0.868	0.01
Stage N2 (%)	46.2 ± 0.3	46.8 ± 0.4	0.5 (−0.5, 1.5)	0.296	0.05
Stage N3 (%)	19.9 ± 0.2	19.5 ± 0.3	−0.4 (−1.2, 0.4)	0.324	0.05
REM (%)	21.9 ± 0.2	21.8 ± 0.2	−0.1 (−0.7, 0.5)	0.829	0.01
AHI (events/h)	15.6 ± 0.4	15.5 ± 0.6	−0.1 (−1.6, 1.4)	0.876	0.01
Awakening index (events/h)^a^	3.6 ± 0.1	3.7 ± 0.1	0.0 (−0.1, 0.3)	0.165	0.07
PLMS index (events/h)^b^	3.5 ± 0.2	3.2 ± 0.3	−0.07 (−0.2, 0.1)	0.358	0.05
Arousal index (events/h)	21.1 ± 0.3	21.6 ± 0.4	0.4 (−0.6, 1.5)	0.410	0.04
Spontaneous arousal index (events/h)	9.5 ± 0.1	9.8 ± 0.2	0.3 (−0.2, 0.8)	0.246	0.06
Respiratory arousal index (events/h)^a^	5.6 ± 0.2	5.7 ± 0.3	0.1 (−0.6, 0.7)	0.916	0.01
RERA index (events/h)^b^	0.7 ± 0.0	0.7 ± 0.0	0.0 (0.0, 0.1)	0.413	0.04
PLMS arousal index (events/h)^b^	1.0 ± 0.1	1.0 ± 0.1	0.0 (−0.1, 0.1)	0.970	0.00

*Note:* Data were analysed by multiple linear regressions with sleep variables as the dependent variables and insomnia symptoms as the independent variable, adjusting for age, sex, current major depressive disorder, current anxiety disorder and psychotropic medication use. Values represent adjusted means ± standard error. The unstandardised beta coefficient (*B*) reflects the adjusted difference between participants with insomnia symptoms and those without (insomnia minus no insomnia). Bold text indicates p < 0.05. Missing values: REM sleep latency (*n* = 8). Complete case analysis was performed in case of missing values.

Abbreviations: AHI, apnea‐hypopnea index; PLMS, periodic limb movements during sleep; REM, rapid eye movement; RERA, respiratory effort‐related arousal; TST, total sleep time; WASO, wake after sleep onset.

^a^ Square‐transformed variables (back‐transformed for presentation).

^b^ Log‐transformed variables (back‐transformed for presentation).

### 3.3 Sleep Microstructure

Participants with insomnia symptoms showed higher ORP values during wake, NREM sleep and N2 sleep, as well as higher ORP‐9 compared with participants without insomnia symptoms. In addition, the insomnia symptoms group showed higher normalised EEG power, higher beta‐1 power, higher beta‐2 power and higher gamma power. Across all significant associations, effect sizes were small (Table 4).

**TABLE 4:** Sleep microstructure variables associated with insomnia symptoms (*n* = 1574).

	No insomnia symptoms (*n* = 1047; 66.5%)	Insomnia symptoms (*n* = 527; 33.5%)	B (95% CI)	*p*	Cohen's *d*
ORP in wake	2.01 ± 0.01	2.03 ± 0.01	0.02 (0.00, 0.05)	**0.023**	0.12
ORP in NREM	0.99 ± 0.01	1.02 ± 0.01	0.03 (0.00, 0.06)	**0.020**	0.12
ORP in N1	1.53 ± 0.01	1.55 ± 0.01	0.02 (0.00, 0.05)	0.101	0.09
ORP in N2	1.04 ± 0.01	1.07 ± 0.01	0.03 (0.00, 0.06)	**0.038**	0.11
ORP in N3	0.58 ± 0.01	0.60 ± 0.01	0.02 (−0.01, 0.04)	0.164	0.08
ORP in REM	1.43 ± 0.01	1.44 ± 0.01	0.01 (−0.03, 0.04)	0.724	0.02
ORP‐9	1.25 ± 0.01	1.30 ± 0.01	0.05 (0.02, 0.08)	**0.003**	0.16
ORP‐ICC^a^	0.84 ± 0.00	0.85 ± 0.00	0.00 (0.00, 0.01)	0.415	0.05
Normalised EEG power	0.90 ± 0.01	0.94 ± 0.01	0.04 (0.00, 0.07)	**0.028**	0.12
Alpha intrusion (%)^b^	2.92 ± 0.08	3.07 ± 0.12	0.04 (−0.04, 0.11)	0.333	0.05
Delta power (μV^2^)	54.65 ± 0.83	52.62 ± 1.14	0.04 (−0.09, 0.01)	0.150	0.08
Theta power (μV^2^)	10.69 ± 0.11	10.53 ± 0.15	−0.01 (−0.04, 0.02)	0.387	0.05
Alpha power (μV^2^)	6.96 ± 0.11	6.93 ± 0.16	0.00 (−0.05, 0.04)	0.889	0.01
Sigma power (μV^2^)^b^	1.27 ± 0.02	1.33 ± 0.03	0.03 (0.00, 0.05)	0.054	0.10
Beta‐1 power (μV^2^)	1.86 ± 0.02	1.97 ± 0.03	0.04 (0.01, 0.06)	**0.007**	0.15
Beta‐2 power (μV^2^)	1.45 ± 0.02	1.58 ± 0.03	0.05 (0.02, 0.08)	**< 0.001**	0.19
Gamma power (μV^2^)^b^	0.71 ± 0.01	0.79 ± 0.03	0.05 (0.01, 0.09)	**0.011**	0.14

*Note:* Data were analysed by multiple linear regressions with sleep variables as the dependent variables and insomnia symptoms as the independent variable, adjusting for age, sex, current major depressive disorder, current anxiety disorder and psychotropic medication use. Values represent adjusted means ± standard error. The unstandardised beta coefficient (B) reflects the adjusted difference between participants with insomnia symptoms and those without (insomnia minus no insomnia). ORP measures and normalised EEG power are unitless variables. Bold text indicates *p *< 0.05. Missing values: normalised EEG power (*n* = 34), ORP in N3 (*n* = 10), ORP in REM (11), ORP‐9 (*n* = 9), ORP‐ICC (*n* = 298). Complete case analysis was performed in case of missing values.

Abbreviations: ICC, interhemispheric coherence; ORP, odds ratio product; ORP‐9, average ORP in the 9 s after an arousal or awakening; REM, rapid eye movement.

^a^ Square‐transformed variables (back‐transformed for presentation).

^b^ Log‐transformed variables (back‐transformed for presentation).

### 3.4 Sensitivity Analyses

Compared to participants who did not undergo PSG (*n* = 2229), those who underwent PSG (*n* = 1718) were less likely to be female, less likely to be current smokers and had higher ESS scores (Table S1).

When analyses were restricted to participants with available PSG data (*N* = 1718), differences in sociodemographic and clinical characteristics were largely similar to those observed in the full sample, except that differences in hypertension and smoking status were no longer statistically significant (Table S2).

In the multivariable model performed in this subsample (*n* = 1718), associations of insomnia symptoms with older age, female sex, fewer years of education and current anxiety disorder remained significant. In this subsample, insomnia symptoms were also associated with an evening chronotype. However, associations with dyslipidaemia, smoking status, excessive alcohol consumption, current major depressive disorder and psychotropic medication use were attenuated and no longer statistically significant (Table S3).

When analyses of sleep macrostructure were restricted to participants with available microstructure data (*n* = 1574), the results regarding TST, WASO and sleep efficiency were unchanged, whereas sleep onset latency was no longer significantly different between the groups (Table S4).

No significant interactions between insomnia symptoms and age or sex were observed for sleep macrostructure. For sleep microstructure, a significant age interaction was observed for gamma power (*p* = 0.008), with higher gamma power observed in participants with insomnia symptoms at the mean age (58.8 years) and at one standard deviation below the mean age (47.4 years), but not at one standard deviation above the mean age (69.6 years). A significant sex interaction was observed for alpha intrusion (*p* = 0.011), with higher alpha intrusion in men with insomnia symptoms but no significant difference among women (Table S5).

## 4 Discussion

In this population‐based sample of Swiss adults, the prevalence of insomnia symptoms was 32.0%, and regular use of hypnotics was reported by 4.2% of the participants. Older age, female sex, fewer years of education, lower likelihood to be a current smoker, dyslipidaemia, excessive alcohol consumption, current major depressive disorder, current anxiety disorder, psychotropic medication use and greater ESS scores were independently associated with insomnia symptoms.

These findings align with another Swiss study reporting a similar prevalence of insomnia symptoms (36%) among adult primary care patients (Maire et al. 2020). Comparable prevalence estimates have also been observed in other European countries (Hafner et al. 2023). Furthermore, the sociodemographic, clinical and psychiatric correlates identified in our study are consistent with prior literature (Xiang et al. 2008).

One unexpected finding was the association between insomnia symptoms and a lower likelihood of being a current smoker. Given that nicotine is a stimulant and has been associated with sleep disruption (Jaehne et al. 2009), this result warrants cautious interpretation. The non‐smoking group included both never‐smokers and former smokers, and it is possible that individuals with insomnia symptoms may have been more likely to quit smoking due to health concerns or medical advice. However, this association was not observed in sensitivity analyses restricted to participants who underwent PSG, suggesting that the finding may reflect limited robustness or residual confounding.

Only five population‐based studies have examined insomnia and PSG‐derived sleep macrostructure. Three Sleep Heart Health Study analyses defined insomnia as ≥ 16 events/month (Pallesen et al. 2014; Baglioni et al. 2014; Ma et al. 2021). These investigations reported reduced sleep efficiency, longer WASO, longer REM latency and altered proportions of NREM sleep stages among individuals with insomnia (Pallesen et al. 2014; Baglioni et al. 2014; Ma et al. 2021). An analysis of the Wisconsin Sleep Cohort used a ≥ 5 events/month threshold for defining insomnia and found short TST and reduced sleep efficiency in participants with insomnia (Lyu et al. 2020). Lucena et al. used data from the Sao Paulo epidemiologic sleep study (EPISONO), defined insomnia using a threshold of ≥ 3 nights/weeks, and adjusted for major confounders; they found lower sleep efficiency, longer sleep onset latency, and longer WASO in insomnia (Lucena et al. 2020). Sleep efficiency was the only measure consistently associated with insomnia across all five studies (Pallesen et al. 2014; Baglioni et al. 2014; Ma et al. 2021; Kang et al. 2022; Lyu et al. 2020), and in the present analysis, suggesting it may be the most robust conventional PSG marker of insomnia in general population samples.

Nevertheless, the differences in sleep macrostructure observed in the present study were small and likely of limited clinical relevance. No group differences emerged for sleep stages, REM latency, or arousal indices. Despite longer WASO, arousal indices did not differ significantly between groups, suggesting that insomnia may involve greater difficulty returning to sleep after awakenings rather than more frequent awakenings. The modest magnitude of the macrostructural differences observed here may partly reflect the population‐based design of the study. Individuals with insomnia symptoms in the general population represent a heterogeneous spectrum of sleep complaints and may differ substantially from patients recruited from specialised sleep clinics, where symptoms are typically more severe and persistent. Additionally, these findings are consistent with the well‐described discrepancy between subjective sleep complaints and objective PSG findings in insomnia, often referred to as subjective–objective sleep discrepancy or sleep state misperception.

In the present study, individuals with insomnia symptoms had higher ORP during wake, NREM sleep, and N2 sleep, as well as higher ORP‐9. These findings are consistent with previous reports in individuals with insomnia (Younes et al. 2021), reflecting generally lighter sleep, higher arousability and slower recovery to deeper sleep following an arousal. The observed alterations in ORP metrics support the hypothesis that insomnia is characterised by increased physiological arousal and reduced sleep stability. Even in this population‐based sample, ORP measures appeared sensitive to subtle differences in sleep depth and arousal dynamics between individuals with and without insomnia symptoms.

Participants with insomnia symptoms also had higher beta and gamma power, which is consistent with reports of elevated fast‐frequency EEG activity in insomnia (Buysse et al. 2008; Lambing et al. 2025; Riedner et al. 2016). Elevated fast‐frequency activity during sleep is often interpreted as reflecting cortical hyperarousal, a central hypothesis in the pathophysiology of insomnia. Relatedly, insomnia was associated with greater alpha intrusion, corroborating previous results and showing the intrusion of wake‐like EEG activity into sleep (Younes et al. 2021). In the present study, however, this difference was observed only in men. Participants with insomnia symptoms also showed higher normalised EEG power, which has previously been associated with accelerated cognitive decline (Younes et al. 2023).

Although several sleep microstructure variables differed significantly between insomnia groups, the magnitude of these differences was small. This may reflect the heterogeneity of sleep physiology among individuals reporting insomnia complaints: some individuals show objective microstructural alterations in sleep, whereas others display sleep patterns more similar to those of good sleepers (Lambing et al. 2025).

We also explored whether the associations between insomnia symptoms and sleep macrostructural and microstructural metrics differed according to age or sex. Overall, interactions were uncommon, suggesting that the relationships between insomnia symptoms and objective sleep measures were largely consistent across demographic groups. The only significant interactions were observed for gamma power with age and for alpha intrusion with sex, indicating that the overall pattern of associations was broadly similar across age groups and between men and women in this population‐based sample.

This study had several limitations. First, our definition of insomnia symptoms did not correspond to the criteria for chronic insomnia disorder, which requires symptom duration ≥ 3 months, evidence of daytime impairment and clinical confirmation. However, to our knowledge, no large‐scale population‐based study using PSG has operationalised chronic insomnia disorder in this way. Although validated instruments such as the Insomnia Severity Index (Bastien et al. 2001) or the Bergen Insomnia Scale (Pallesen et al. 2008) may provide a more comprehensive assessment of insomnia symptom severity, these scales were not available in the present dataset, which may have limited the precision with which insomnia symptoms were characterised.

Second, polysomnographic recordings were obtained on average 1 year after the assessment of insomnia symptoms. Given that insomnia symptoms and hypnotic medication use may fluctuate over time, we cannot be certain that participants classified as having insomnia symptoms at baseline experienced similar symptoms at the time of PSG. Along the same lines, even if insomnia symptoms were still present, they may vary from night to night, and individuals with insomnia may therefore experience nights of relatively normal sleep. Because PSG was recorded during a single night, it may not have captured a representative night of sleep for all participants with insomnia symptoms. Single‐night PSG assessments are particularly challenging in insomnia research, as they may reflect either an atypically good or poor night. Future studies using multiple nights of objective sleep recording or combining PSG with concurrent subjective sleep reports could better capture typical sleep patterns in individuals with insomnia.

Third, the study sample included only middle‐aged and older adults, with no participants younger than 40 years. As insomnia symptoms and their objective correlates may differ across the lifespan, the generalisability of these findings to younger populations is limited.

In conclusion, insomnia symptoms were common in this Swiss population‐based sample and were associated with a broad set of sociodemographic, cardiometabolic and psychiatric correlates. Conventional sleep macrostructural metrics differed only modestly between groups, whereas sleep microstructural measures appeared more consistently associated with insomnia symptoms, although with small effect sizes. These findings support the view that objective insomnia‐related sleep alterations are subtle and heterogeneous in general population samples. Sleep microstructural markers may nevertheless be promising tools for refining insomnia phenotyping in future research using prospective population‐based cohorts.

## Author Contributions

**Geoffroy Solelhac:** writing – review and editing. **Pedro Marques‐Vidal:** data curation, writing – review and editing. **Marie‐Pierre Françoise Strippoli:** data curation, writing – review and editing. **Magdy Younes:** writing – review and editing, methodology, data curation. **Théo Imler:** writing – review and editing. **Isabel Ericson:** conceptualization, formal analysis, visualization, writing – original draft. **Peter Vollenweider:** writing – review and editing, data curation. **Martin Preisig:** funding acquisition, data curation, writing – review and editing. **Raphaël Heinzer:** funding acquisition, data curation, writing – review and editing. **Nicola A. Marchi:** conceptualization, supervision, visualization, writing – original draft. **Kari Lambing:** writing – review and editing.

## Funding

The CoLaus|PsyCoLaus and HypnoLaus studies were supported by GlaxoSmithKline, University of Lausanne (Faculty of Biology and Medicine), Swiss National Science Foundation (grants 3200B0‐105993, 3200B0‐118308, 33CSCO‐122661, 33CS30‐139468, 33CS30‐148401, 33CS30‐177535, 3247730_204523 and 320030_220190), Swiss Personalized Health Network (grant 2018DRI01), Leenaards Foundation and Ligue Pulmonaire Vaudoise. The funders had no role in the design of the study; in the collection, analyses, or interpretation of data; in the writing of the manuscript; or in the decision to publish the results.

## Conflicts of Interest

P.M.V. reports receiving grants from ResMed. The other authors declare no conflicts of interest.

## Supporting information


**Data S1:** Supporting Information.
**Table S1:** Characteristics of the participants who had PSG versus those who did not have PSG.
**Table S2:** Characteristics of participants with and without insomnia symptoms in the sample who had PSG (*n* = 1718).
**Table S3:** Multivariable associations of sociodemographic and clinical characteristics with insomnia symptoms in the sample who had a PSG (*n* = 1718).
**Table S4:** Sleep macrostructure variables associated with insomnia symptoms in the sample with microstructure measures available (*n* = 1574).
**Table S5:** Stratified results of the significant interactions between age or sex and insomnia symptoms regarding sleep microstructure (*n* = 1574).
**Figure S1:** Flowchart of the study population.

## Data Availability

The data of CoLaus|PsyCoLaus study used in this article cannot be fully shared as they contain potentially sensitive personal information on participants. According to the Ethics Committee for Research of the Canton of Vaud, sharing these data would be a violation of the Swiss legislation with respect to privacy protection. However, coded individual‐level data that do not allow researchers to identify participants are available upon request to researchers who meet the criteria for data sharing of the CoLaus|PsyCoLaus Datacenter (Lausanne University Hospital—CHUV, Lausanne, Switzerland). Any researcher affiliated to a public or private research institution who complies with the CoLaus|PsyCoLaus standards can submit a research application to research.colaus@chuv.ch or research.psycolaus@chuv.ch. Proposals will be evaluated by the Scientific Committee of the CoLaus|PsyCoLaus studies. Detailed instructions for gaining access to the CoLaus|PsyCoLaus data used in this study are available at www.colaus‐psycolaus.ch/professionals/how‐to‐collaborate.
