# Supplementary material for: Insomnia Symptoms in a Swiss Population‐Based Cohort: Prevalence, Clinical Correlates and Polysomnographic Alterations
Source: J Sleep Res. 2026 Jun 4;35(5):e70376. doi: 10.1111/jsr.70376 (PMC13619071; doi:10.1111/jsr.70376)
Supplement: Supplementary file 1 [file JSR-35-e70376-s001.docx]

**SUPPLEMENTARY MATERIAL**

**Insomnia symptoms in a Swiss population-based cohort: prevalence, clinical correlates, and polysomnographic alterations**

**SUPPLEMENTARY METHODS**

**Ethics**

The institutional Ethics Committee of the University of Lausanne (later replaced by the Ethics Commission of the Canton of Vaud ([www.cer-vd.ch)](rewritten://cbc12e76-483f-40c6-812f-3747d57d347e) approved the CoLaus|PsyCoLaus study at baseline (reference 16/03; 134-03, 134-05bis, 134-05-2 to 5, addenda 1 to 4). Ethical approval was subsequently renewed for each follow-up: the first follow-up (reference 33/09; 239/09), the second (reference 26/14; 239/09 addendum 2), the third (PB_2018-00040; 239/09 addenda 3 to 4), and the fourth (PB_2018_0038; 239/09). The study was conducted in accordance with the Declaration of Helsinki and its amendments, as well as applicable Swiss legislation. All participants provided written informed consent prior to participation.

**Polysomnography**

Polysomnography (PSG) was performed using a standardized protocol by trained technicians at the Center for Investigation and Research in Sleep, Lausanne University Hospital, Switzerland. Between 5 PM and 8 PM, participants were equipped with a portable PSG device (Titanium, Embla Flaga, Reykjavik, Iceland). Recordings were conducted overnight in each participant’s home and followed the American Academy of Sleep Medicine guidelines for signal acquisition and electrode placement.^1^ A total of 18 channels were recorded, including six EEG channels (F3, F4, C1, C2, O1, O2), two electrooculography channels, three electromyography channels (one submental and two anterior tibialis), one electrocardiogram channel, nasal pressure sensors, thoracic and abdominal belts for respiratory effort, pulse oximetry, body position sensors, and pulse rate monitoring. All recordings were visually scored by two trained sleep technicians using Somnologica software (version 5.1.1, Embla Flaga) and reviewed by a board-certified sleep physician. A second physician performed random quality checks to ensure consistency and accuracy.

**Missing data**

Missing values in sociodemographic, clinical, and self-reported sleep questionnaire variables were handled with complete case analysis for descriptive statistics. For the other analyses, we used multiple imputations by chained equations in R. Specifically, imputation was performed (i) for all predictors included in multivariable logistic regression examining correlates of insomnia symptoms, and (ii) for one covariate (current major depressive disorder) included in linear regression models. Binary variables were imputed using logistic regression, categorical variables with multinomial regression, and continuous variables with predictive mean matching. Twenty imputed datasets were generated under the missing-at-random assumption, and results were pooled using Rubin’s rules. All PSG variables were complete, with the exception of REM sleep onset latency, which was undefined for eight participants who did not exhibit REM sleep on the night of PSG recording; these cases were excluded from analyses involving this outcome (complete case analysis).

**SUPPLEMENTARY TABLES**

**Table S1.** Characteristics of the participants who had PSG versus those who did not have PSG

|  | **PSG (n = 1,718)** | **No PSG (n = 2,229)** | **Test** | **P** |
| --- | --- | --- | --- | --- |
| Age, years | 57.4 ± 10.5 | 57.5 ± 10.5 | t = 0.3 | 0.746 |
| Females, n (%) | 847 (49.3) | 1,205 (54.1) | x^2^ = 8.6 | **0.003** |
| Education, years | 13.8 ± 4.1 | 13.6 ± 4.2 | t = 1.6 | 0.098 |
| BMI, kg/m^2^ | 26.1 ± 4.3 | 25.9 ± 4.6 | t = 1.1 | 0.276 |
| Hypertension, n (%) | 707 (41.2) | 892 (40.1) | x^2^ = 0.4 | 0.517 |
| Diabetes, n (%) | 156 (9.1) | 218 (9.8) | x^2^ = 0.5 | 0.461 |
| Dyslipidemia, n (%) | 450 (26.2) | 623 (28.1) | x^2^ = 1.6 | 0.199 |
| Current smoker, n (%) | 299 (17.4) | 524 (23.5) | x^2^ = 21.3 | **<0.001** |
| Excessive alcohol consumption, n (%) | 251 (14.6) | 358 (16.1) | x^2^ = 1.5 | 0.228 |
| Current major depressive disorder, n (%) | 102 (6.4) | 103 (5.9) | x^2^ = 0.3 | 0.606 |
| Current anxiety disorder, n (%) | 84 (5.4) | 98 (6.1) | x^2^ = 0.6 | 0.430 |
| Hypnotics, n (%) | 75 (4.4) | 91 (4.1) | x^2^ = 0.2 | 0.661 |
| Psychotropic medication, n (%) | 193 (11.2) | 234 (10.5) | x^2^ = 0.5 | 0.492 |
| ESS score, points | 6.1 ± 3.8 | 5.3 ± 3.6 | t = 6.6 | **<0.001** |
| MEQ chronotype |  |  | x^2^ = 0.3 | 0.850 |
| Intermediate, n (%) | 1,395 (89.5) | 1,755 (89.1) |  |  |
| Evening, n (%) | 148 (9.5) | 193 (9.8) |  |  |
| Morning, n (%) | 15 (1.0) | 22 (1.1) |  |  |

Data are presented as mean ± standard deviation or number of participants (%). Data were analyzed using independent samples t-tests (t) and chi-squared tests (x^2^). Missing values: BMI (n = 36), hypertension (n = 8), diabetes (n = 13), current smoker (n = 6), current major depressive disorder (n = 585), current anxiety disorder (n = 805), ESS score (n = 189), MEQ (n = 419). Complete case analysis was performed. Bold text indicates p<0.05. Abbreviations: BMI = body mass index; ESS = Epworth Sleepiness Scale; MEQ = Morningness-Eveningness Questionnaire.

**Table S2.** Characteristics of participants with and without insomnia symptoms in the sample who had PSG (n = 1,718)

|  | **No insomnia symptoms (n = 1,144; 66.6%)** | **Insomnia symptoms (n = 574; 33.4%)** | **Test** | **P** |
| --- | --- | --- | --- | --- |
| Age, years | 56.8 ± 10.5 | 58.7 ± 10.3 | t = 3.7 | **<0.001** |
| Female, n (%) | 530 (46.3) | 317 (55.2) | x^2^ = 11.8 | **0.001** |
| Education, years | 14.1 ± 4.2 | 13.4 ± 3.7 | t = 3.6 | **<0.001** |
| BMI, kg/m^2^ | 26.0 ± 4.3 | 26.2 ± 4.4 | t = 0.8 | 0.443 |
| Hypertension, n (%) | 457 (40.0) | 250 (43.6) | x^2^ = 1.8 | 0.176 |
| Diabetes, n (%) | 102 (8.9) | 54 (9.4) | x^2^ = 0.1 | 0.810 |
| Dyslipidemia, n (%) | 297 (26.0) | 153 (26.7) | x^2^ = 0.1 | 0.810 |
| Current smoker, n (%) | 90 (15.7) | 209 (18.3) | x^2^ = 1.7 | 0.194 |
| Excessive alcohol consumption, n (%) | 163 (14.2) | 88 (15.3) | x^2^ = 0.3 | 0.598 |
| Current major depressive disorder, n (%) | 55 (5.1) | 47 (8.9) | x^2^ = 8.1 | **0.005** |
| Current anxiety disorder, n (%) | 46 (4.4) | 38 (7.5) | x^2^ = 5.5 | **0.019** |
| Psychotropic medication, n (%) | 106 (9.3) | 87 (15.2) | x^2^ = 12.7 | **<0.001** |
| ESS score, points | 6.1 ± 3.7 | 6.1 ± 4.0 | t = 0.1 | 0.956 |
| MEQ chronotype |  |  | x^2^ = 5.2 | 0.075 |
| Intermediate, n (%) | 944 (89.9) | 451 (88.8) |  |  |
| Evening, n (%) | 100 (9.5) | 48 (9.4) | 6 (0.6) | 9 (1.8) |
| Morning, n (%) | 6 (0.6) | 9 (1.8) |  |  |

Data are presented as mean ± standard deviation or number of participants (%). Data were analyzed using independent samples t-tests (t) and chi-squared tests (x^2^). Missing values: BMI (n = 10), hypertension (n = 2), diabetes (n = 1), current smoker (n = 4), current major depressive disorder (n = 112), current anxiety disorder (n = 170), ESS score (n = 59), MEQ (n = 160). Complete case analysis was performed. Bold text indicates p <0.05. Abbreviations: BMI = body mass index; ESS = Epworth Sleepiness Scale; MEQ = Morningness-Eveningness Questionnaire.

**Table S3.** Multivariable associations of sociodemographic and clinical characteristics with insomnia symptoms in the sample who had a PSG (n = 1,718)

|  | **Unadjusted OR** | **P** | **Adjusted OR (95% CI)** | **P** |
| --- | --- | --- | --- | --- |
| Age (per 5-year increase) | 1.09 (1.04, 1.15) | **<0.001** | 1.10 (1.04, 1.16) | **0.001** |
| Female sex | 1.43 (1.17,1.75) | **0.001** | 1.38 (1.10, 1.74) | **0.004** |
| Education (per 1-year increase) | 0.96 (0.93, 0.98) | **0.001** | 0.97 (0.94, 0.99) | **0.010** |
| BMI (per 1-kg/m^2^ increase) | 1.01 (0.99, 1.03) | 0.472 | 1.00 (0.97, 1.03) | 0.999 |
| Hypertension | 1.15 (0.94, 1.41) | 0.172 | 1.01 (0.79, 1.28) | 0.952 |
| Diabetes | 1.06 (0.75, 1.49) | 0.756 | 0.90 (0.62, 1.32) | 0.591 |
| Dyslipidemia | 1.03 (0.82, 1.30) | 0.783 | 1.13 (0.88, 1.45) | 0.329 |
| Smoking | 0.83 (0.63, 1.08) | 0.168 | 0.78 (0.59, 1.04) | 0.088 |
| Excessive alcohol consumption | 1.09 (0.82, 1.44) | 0.547 | 1.23 (1.91, 1.65) | 0.180 |
| Current major depressive disorder | 1.79 (1.20, 2.66) | **0.004** | 1.51 (0.98, 2.31) | 0.059 |
| Current anxiety disorder | 1.72 (1.11, 2.66) | **0.015** | 1.67 (1.05, 2.64) | **0.029** |
| Psychotropic medication | 1.75 (1.29, 2.37) | **<0.001** | 1.39 (1.00, 1.93) | 0.051 |
| ESS score (per 1-point increase) | 1.00 (0.97, 1.03) | 0.899 | 1.01 (0.99, 1.04) | 0.310 |
| MEQ chronotype |  |  |  |  |
| Intermediate (ref.) |  |  |  |  |
| Evening | 2.98 (1.06, 8.34) | **0.038** | 3.29 (1.14, 9.47) | **0.027** |
| Morning | 0.99 (0.69, 1.42) | 0.955 | 0.97 (0.67, 1.40) | 0.876 |

Data were analyzed using binomial logistic regression with insomnia symptoms as the dependent variable. Unadjusted OR were calculated for each predictor separately, whereas adjusted OR were derived from a multivariable model including all predictors listed in the table. Missing data were handled using multiple imputation. Results are presented as odds ratios (OR) with 95% confidence intervals (CI). Abbreviations: BMI = body mass index; ESS = Epworth Sleepiness Scale; MEQ = Morningness-Eveningness Questionnaire.

**Table S4.** Sleep macrostructure variables associated with insomnia symptoms in the sample with microstructure measures available (n = 1,574)

|  | **No insomnia symptoms**  **(n = 1,047; 66.5%)** | **Insomnia symptoms  (n = 527; 33.5%)** | **B (95% CI)** | **P** | **Cohen’s *d*** |
| --- | --- | --- | --- | --- | --- |
| TST (min) | 402.9 ± 2.1 | 392.9 ± 3.0 | -10.0 (-17.3, -2.8) | **0.007** | 0.15 |
| Sleep onset latency (min)** | 9.8 ± 0.3 | 10.9 ± 0.5 | 0.1 (0.0, 0.2) | 0.095 | 0.09 |
| REM latency (min)* | 86.7 ± 1.5 | 88.8 ± 2.2 | 0.1 (-0.2, 0.4) | 0.431 | 0.04 |
| Sleep efficiency (%) | 85.3 ± 0.3 | 83.3 ± 0.4 | -2.0 (-3.0, -1.0) | **<0.001** | 0.21 |
| WASO (min) | 71.4 ± 1.5 | 80.6 ± 2.2 | 9.2 (3.9, 14.6) | **0.001** | 0.18 |
| Stage N1 (%) | 11.9 ± 0.2 | 11.9 ± 0.3 | -0.02 (-0.7, 0.7) | 0.960 | 0.00 |
| Stage N2 (%) | 46.2 ± 0.3 | 46.8 ± 0.4 | 0.7 (-0.4, 1.7) | 0.220 | 0.01 |
| Stage N3 (%) | 19.9 ± 0.2 | 19.5 ± 0.4 | -0.4 (-1.3, 0.4) | 0.317 | 0.05 |
| REM (%) | 22.0 ± 0.2 | 21.8 ± 0.3 | -0.2 (-0.8, 0.4) | 0.515 | 0.04 |
| AHI (events/h) | 15.8 ± 0.5 | 15.6 ± 0.7 | -0.2 (-1.8, 1.4) | 0.764 | 0.02 |
| Awakening index (events/h)* | 3.6 ± 0.1 | 3.7 ± 0.1 | 0.0 (0.0, 0.1) | 0.190 | 0.07 |
| PLMS index (events/h)** | 3.4 ± 0.2 | 3.2 ± 0.3 | -0.1 (-0.2, 0.1) | 0.442 | 0.04 |
| Arousal index (events/h) | 21.3 ± 0.3 | 21.8 ± 0.5 | 0.5 (-0.6, 1.6) | 0.395 | 0.05 |
| Spontaneous arousal index (events/h) | 9.6 ± 0.1 | 9.9 ± 0.2 | 0.3 (-0.2, 0.8) | 0.221 | 0.07 |
| Respiratory arousal index (events/h)* | 5.7 ± 0.2 | 5.7 ± 0.3 | 0.0 (-0.2, 0.1) | 0.842 | 0.01 |
| RERA index (events/h)** | 0.7 ± 0.0 | 0.7 ± 0.0 | 0.0 (0.0, 0.1) | 0.301 | 0.06 |
| PLMS arousal index (events/h)** | 1.0 ± 0.1 | 1.0 ± 0.1 | -0.0 (-0.1, 0.1) | 0.982 | 0.00 |

Data were analyzed by multiple linear regressions with sleep variables as the dependent variables and insomnia symptoms as the independent variable, adjusting for age, sex, current major depressive disorder, current anxiety disorder, and psychotropic medication use. Values represent adjusted means ± standard error. The unstandardized beta coefficient (B) reflects the adjusted difference between participants with insomnia symptoms and those without (insomnia minus no insomnia). Missing values: REM sleep latency (n = 5). Complete case analysis was performed in case of missing values. *Square-transformed variables (back-transformed for presentation). **Log-transformed variables (back-transformed for presentation). Abbreviations: AHI = apnea-hypopnea index; PLMS = periodic limb movements during sleep; RERA = respiratory effort-related arousal; TST = total sleep time; WASO = wake after sleep onset; REM = rapid eye movement.

**Table S5.** Stratified results of the significant interactions between age or sex and insomnia symptoms regarding sleep microstructure (n = 1,574)

|  | **No insomnia symptoms**  **(n = 1,047; 66.5%)** | **Insomnia symptoms**  **(n = 527; 33.5%)** | **B (95% CI)** | **P** | **Cohen’s *d*** |
| --- | --- | --- | --- | --- | --- |
| Alpha intrusion (%)** |  |  |  |  |  |
| Males | 2.26 ± 0.08 | 2.75 ± 0.15 | 0.14 (0.05, 0.23) | **0.003** | 0.23 |
| Females | 3.95 ± 0.18 | 3.61 ± 0.21 | -0.07 (-0.19, 0.05) | 0.238 | 0.09 |
| Gamma power (µV²)** |  |  |  |  |  |
| Age -1 SD (47.4 years) | 0.65 ± 0.02 | 0.82 ± 0.04 | 0.17 (0.08, 0.26) | **<0.001** | 0.28 |
| Mean age (58.8 years) | 0.72 ± 0.02 | 0.80 ± 0.03 | 0.08 (0.02, 0.15) | **0.009** | 0.14 |
| Age +1 SD (69.6 years) | 0.79 ± 0.03 | 0.79 ± 0.04 | 0.00 (-0.09, 0.09) | 0.981 | 0.00 |

Data were analyzed by multiple linear regressions with sleep variables as the dependent variables and insomnia symptoms as the independent variable, adjusting for age (only for alpha intrusion), sex (only for gamma power), current major depressive disorder, current anxiety disorder, and psychotropic medication use. Values represent adjusted means ± standard error. The unstandardized beta coefficient (B) reflects the adjusted difference between participants with insomnia symptoms and those without (insomnia minus no insomnia). **Log-transformed variables (back-transformed for presentation). Abbreviations: SD = standard deviation.

**SUPPLEMENTARY FIGURES**

**Figure S1.** Flowchart of the study population

Abbreviations: PSG = polysomnography; PSQI = Pittsburgh Sleep Quality Index; RLS = restless legs syndrome.

# **SUPPLEMENTARY REFERENCES**

1.	Berry, R. B. *et al.* Rules for Scoring Respiratory Events in Sleep: Update of the 2007 AASM Manual for the Scoring of Sleep and Associated Events: Deliberations of the Sleep Apnea Definitions Task Force of the American Academy of Sleep Medicine. *J. Clin. Sleep Med.* **08**, 597–619 (2012).
